# Orthotopic and Heterotopic Murine Models of Pancreatic Cancer Exhibit Different Immunological Microenvironments and Different Responses to Immunotherapy

**DOI:** 10.3389/fimmu.2022.863346

**Published:** 2022-07-07

**Authors:** Jin Wang, Xingchen Liu, Junsong Ji, Jianhua Luo, Yuanyu Zhao, Xiaonan Zhou, Jianming Zheng, Meng Guo, Yanfang Liu

**Affiliations:** ^1^ Department of pathology, Changhai Hospital, Navy Medical University, Shanghai, China; ^2^ Institute of Organ Transplantation, Changzheng Hospital, Navy Medical University, Shanghai, China; ^3^ National Key Laboratory of Medical Immunology & Institute of Immunology, Navy Medical University, Shanghai, China; ^4^ Department of Anesthesiology, Changzheng Hospital, Navy Medical University, Shanghai, China

**Keywords:** orthotopic PDAC animal model, immunological checkpoint blockade, CAR-T, MDSC, NKG2D, NKG2DL

## Abstract

For decades, tumor-bearing murine models established using tumor cell lines have been the most commonly used models to study human cancers. Even though there are several studies reported that implant sites caused disparities in tumor behaviors, few of them illuminated the positional effect on immunotherapy. Herein, we describe surgical techniques for a novel orthotopic implantation of syngeneic pancreatic ductal adenocarcinoma (PDAC) tissue slices. This method has a high success modeling rate and stable growth kinetics, which makes it useful for testing novel therapeutics. Pathological examination indicated that the orthotopic tumor displayed poor vascularization, desmoplastic stromal reaction, and a highly immunosuppressive tumor microenvironment. This unique microenvironment resulted in limited response to PD1/CTLA4 blockade therapy and anti-MUC1 (αMUC1) CAR-T transfer treatment. To reverse the suppressive tumor microenvironment, we developed gene modified T-cells bearing a chimeric receptor in which activating receptor NKG2D fused to intracellular domains of 4-1BB and CD3ζ (NKG2D CAR). The NKG2D CAR-T cells target myeloid-derived suppressor cells (MDSCs), which overexpress Rae1 (NKG2D ligands) within the TME. Results indicated that NKG2D CAR-T cells eliminated MDSCs and improved antitumor activity of subsequently infused CAR-T cells. Moreover, we generated a bicistronic CAR-T, including αMUC1 CAR and NKG2D CAR separated by a P2A element. Treatment with the dual targeted bicistronic CAR-T cells also resulted in prolonged survival of orthotopic model mice. In summary, this study describes construction of a novel orthotopic PDAC model through implantation of tissue slices and discusses resistance to immunotherapy from the perspective of a PDAC microenvironment. Based on the obtained results, it is evident that elimination MDSCs by NKG2D CAR could rescue the impaired CAR-T cell activity.

## 1 Introduction

Pancreatic cancer, which has nearly identical incidence and mortality rates, is the leading cause of cancer-related death with a poor prognosis (1, 2). Pancreatic ductal adenocarcinoma (PDAC), the most common pathological type of pancreatic cancer, originates from the ductal cells of the exocrine gland and is characterized by extensive stroma reaction (3). This extensive desmoplastic stroma is believed to create a substantial barrier to perfusion of chemotherapeutics, thereby restricting the efficacy of chemotherapy. Studies have shown that even administration of more intensified chemotherapy regimens, such as modified FOLFIRINOX, only results in modest improvement on the survival rates of PDAC patients (4). Therefore, immunotherapy might constitute a more attractive approach.

In recent years, immunotherapeutic treatment has re-emerged as a promising therapeutic choice in oncology. Notably, immunotherapy seeks to eliminate cancer through both active and passive approaches. For example, the immune checkpoint blockade (ICB) is a typical active approach that facilitates the lysis of tumor cells by reactivating immunologic actions (5), whereas chimeric antigen receptor modified T-cell (CAR-T) is a passive approach which can directly lyse target cells through CAR signaling (6). However, although ICB and CAR-T treatments are highly effective in inhibiting tumor process in PDAC models (7, 8), recent studies have proven that they are clinically ineffective (9, 10).

It is worth noting that ideal animal models may help to bridge the chasm that separates clinical application from experimental findings. For example, a spontaneous PDAC model (KC/KPC), which mimics the microenvironment of human disease, also did not respond to CTLA4 and PD-L1 blockades (11). Conversely, complete responses to ICB treatment were observed in mice subcutaneously engrafted with tumor grafts from KPC mice (8). Moreover, one study demonstrated that tumor xenografts undergo significant copy number alteration (CNA) changes in human cancers (12). However, although subcutaneous tumorigenesis using immunodeficient mice might be the most common model in oncology research, with advantages of approachable manipulation and real-time observation of tumor kinetics, these evidences might weaken the reliability of results obtained from heterotopic PDAC model in the evaluation of immunotherapy. It should also be noted that KC/KPC mice are not a perfect model for immunotherapy research for several reasons. First, tumor formation in KPC mice usually creates several months of latency. Second, consistencies of tumor size in this model are not satisfactory, which brings barriers to assay therapeutic effect. Third, there is a lack of real-time visualization of spontaneous tumors.

An alternative approach is *in situ* tumorigenesis *via* injection of syngeneic PDAC cell line into the pancreatic capsule. Despite this model having a short latency, its success modeling rate remains modest. Notably, the thickness of the murine pancreas, unlike the primate pancreas, is only a few millimeters. Thus, *in situ* injection in murine pancreas capsule is highly susceptible to peritoneal dissemination and subtle differences in injection location often cause unreliable growth kinetics. Although several groups have published their experiences with *in situ* tumor implantation surgical technique (13, 14), the studies lack technical details. In this study, we provide a detailed technical analysis for PDAC modeling using tumor tissue slices as “seeds”. We also describe the differences in the tumor microenvironment between orthotopic and heterotopic tumors, which explain the mechanism of limited response of ICB and CAR-T transfer. Furthermore, based on the above findings, we developed a bicistronic CAR-T expressing anti-MUC1 CAR and NKG2D CAR to simultaneously eliminate myeloid-derived suppressor cells (MDSCs) and cancer cells, thereby enhancing tumor regression.

## 2 Materials and Methods

### 2.1 Animal Study

C57 and nude mice were obtained from Cavens Experimental Animal Company (Changzhou, China). NOD/ShiLtJGpt-*Prkdc^em26Cd52^Il2rg^em26Cd22^*/Gpt (NCG) mice were purchased from Gempharmatech Inc. (Nanjing, China). All mice were housed in specific pathogen-free conditions and maintained under a 12-hour light-dark cycle at 23°C, with ad libitum access to water and standard rodent diet. All animal experiments were approved by the Scientific Investigation Board of Navy Medical University (Shanghai, China).

### 2.2 Lentivirus Preparation and Cell Construction

The human MUC1 (NM_001018016.1) coding sequence was cloned form commercial plasmid (SinoBiological, HG12123). The mice Rae-1 coding sequence (NM_198193.3) was synthesized by Beijing TSINGKE Biotech. The extracellular (EC) and transmembrane (TM) regions of mice NKG2D (NM_001083322.2) were cloned form commercial plasmid (SinoBiological, MG57340). scFv sequence of CD19 was from patent US20210395364A1 in VL-(G_4_S)_3_-VH form. scFv sequence of MUC1 was from patent US10239950B2 in VL-(G_4_S)_3_-VH form.

Lentivirus were generated using Lenti-X 293T cell line and packaging plasmid vectors. Twenty-four hours before transfection, 2×10^7^ Lenti-X 293T cells were seeded in a 150-mm culture dish. Next, CAR-expression lentivirus vectors (pRRLSIN-αCD19/BBZ or pRRLSIN-αMUC1/BBZ or pRRLSIN-NKG2D/BBZ or pRRLSIN-αMUC1/BBZ-P2A- NKG2D/BBZ) or MUC1-expression lentivirus vector (pLVX-MUC1) or RAE1-expression lentivirus vector (pLVX-Rae1) were transfected into packaging cells with psPAX2 and pMD2.G envelop plasmids, at a ratio of 4:3:1, using calcium-phosphate kit (ViralTherapy, R001) in accordance with the manufacturer’s protocol. Supernatants were harvested after 48 and 72 h post-transfection and then they were concentrated by ultracentrifugation for 2.5 h at 82,700g at 4°C. Precipitated lentiviral particles were resuspended in Opti-MEM medium (Solarbio, 31985070). Lentiviral titer was determined using QuickTiter Lentivirus Quantitation Kit (Cell Biolabs, VPK-107) following the manufacturer’s instructions.

Establishment of MUC1 stable expressing Panc02 cells: Murine Panc02 cells were obtained from the American Type Culture Collection (ATCC) and cultured in DMEM medium (BasalMedia, L110KJ) supplemented with 10% FBS (Corning, 35-081-CV). Stable cell lines were then established using lentivirus. Briefly, Panc02 cells were seeded into 24-well plates at 1×10^5^ cells/well, followed by infection with 2mg/mL polybrene (Yeasen, 40804ES76) at MOI=10. Finally, single clones were obtained by FACS sorting of MUC1-positive cells after labelling by anti-MUC1-APC antibody (Biolegend, 355608).

Establishment of Rae1 stable expressing CHO cells: CHO cells were gifted by Mr. Huashun Li from ATCGcell (Suzhou, China) and cultured in SMM CHO-SI serum-free medium (Sinobiological, MCHOSI). Stable cell lines were established using lentivirus. Briefly, CHO cells were seeded into 24-well plates at 1×10^5^ cells/well and then infected with 5 mg/mL polybrene at MOI=5. Single clones were obtained by FACS sorting of Rae1-positive cells after labelling by anti-Rae1-APC antibody (R&D, FAB17582A).

Establishment of CAR-T cells: CAR-T cells were generated by infecting activated T cells with CAR-encoding lentivirus. Briefly, spleens were first harvested from sacrificed C57 mice. T cells were enriched from splenocytes with a T cell isolation kit (Miltenyi, 130-095-130), followed by activation with CD3/CD28 beads (Miltenyi, 130-093-627) and culturing in RPMI-1640 medium supplemented with 30ng/mL IL-2 (Novoprotein, GMP-CD66). After 72 h, T cells were transduced with lentivirus at MOI=5 by 1200×g centrifugal inoculation for 2 h at 25°C in fresh media containing 1% Lentiviral Transduction Enhancer (WeizhenBio, FH880805). The transduced T cells were subsequently washed and cultured in full medium with 30ng/mL IL-2 for 7~10 days. Gene-transfer was estimated by the percentage of FLAG-tag+ (αMUC1-CAR) or Myc-tag+ (NKG2D-CAR) T cells detected by FACS (anti-FLAG (Biolegend, 637318); anti-Myc (Biolegend, 626805)). Notably, CAR-expressing T cells were used immediately after expansion.

### 2.3 Surgical Procedures

All animals were anesthetized with isoflurane (induction: 5%, maintenance: 2.5%). Anesthesia levels were evaluated regularly throughout the operation. The skin at the incision site was sterilized with three scrubs of povidone iodine and one scrub of 75% alcohol. Sterile practices were followed throughout the surgical procedure. The tumor size was calculated as follows: tumor size (cm^3^) = ½ (length × width^2^). Notably, mice were euthanized when the tumor burden was >3.0cm^3^. Overall survival was calculated from the date of tumor engraftment to the date of the last observation or death.

#### 2.3.1 Precision-cutting of Tissue Slices

To obtain the tumor graft, Panc02 cells were subcutaneously inoculated in 6-week-old nude mice (2 × 10^7^ cells in each mouse). Tumors were allowed to grow for 2-3 weeks until they were approximately 8~10 mm in diameter. Next, tumor samples were obtained and immediately transferred into ice-cold UW (University of Wisconsin) preservation solution. Each tumor was then wrapped in 5% low-melt agar and quickly sectioned using a tissue slicer at a thickness of 500μm. The obtained sections were quickly transferred to ice-cold UW solution, and the implantation process was finished within 12 h after surgery.

#### 2.3.2 Tissue Slices Transplantation (TST) method

After disinfection and sterilization, ophthalmic scissors were used to cut a 1 cm port above the spleen. Atraumatic forceps were then used to gently squeeze out the pancreas tail with spleen. The capsule of the pancreas was opened with blunt dissection. Next, the open edge of the capsule was gently lifted and a tumor slice was carefully inserted into the subcapsular space. The graft was fitted to the pancreas with 2μL of Vetbond (3M, 1469SB), followed by reinforcement using 5-0 absorbable sutures. Finally, the spleen and pancreas were incorporated into the abdominal cavity and the abdomen was closed.

#### 2.3.3 Orthotopic Injection (OI) Method

The pancreas was exposed following the same steps described above. Before injection, the injection areas were warmed using 37°C saline. Next, 3×10^6^ Panc02 cells resuspended in 30μL Matrigel (Yeasen, 40134ES08) were immediately administered into the pancreas capsule. Before needle drawing, a heating lamp was used to heat the injection site to promote solidification of the Matrigel. Finally, the spleen and pancreas were incorporated into the abdominal cavity and the abdomen was closed.

### 2.4 Hematoxylin and Eosin (H&E) Staining and Immunohistochemistry (IHC)

H&E staining and Immunohistochemistry were performed as previously described (15, 16). Briefly, tumor specimens were first fixed with 10% formalin for 24 h and 70% ethanol for 12 h prior to embedding in paraffin and sectioning. Tissue sections were then deparaffinized in xylene and rehydrated in graded ethanol.

For H&E staining, after staining with hematoxylin for 5 minutes, sections were stained with eosin solution for 30 seconds. Next, sections were dehydrated and mounted using neutral gum.

For IHC, heat-induced antigen unmasking was performed in a 10mM citrate buffer and then blocked with 1% BSA for 1 h at room temperature. Sections were incubated with primary antibodies at 4°C overnight at an ideal dilution in a humidified chamber. On the next day, sections were incubated with secondary antibodies for 30 minutes at room temperature (Zsbio, pv8000), followed by detection using the DAB detection kit (OriGene Technologies, ZLI-9017). The following primary antibodies were used: anti-Ki67 (Abcam, ab15580; 1:100), anti-F4/80 (Cst,70076; 1:200), anti-CD20 (Abcam, ab64088; 1:100), anti-CD4(Abcam, ab183685; 1:100), anti-CD8 (Abcam, ab228965; 1:100), anti-Collagen type IV (Affinity, af0510; 1:100), anti-CK7 (PTG, 17514-1-Ap; 1:100), anti-CD34 (Abcam, ab81289; 1:100), and anti-Ly6G (Invitrogen, 2335909; 1:100).

### 2.5 Cytotoxicity Assays

CAR-T to tumor cellcytotoxicity assays were determined by the extracellular release of lactate dehydrogenase (LDH) using a CytoTox96 cytotoxicity assay kit (Promega, G1780) according to the manufacturer’s instructions. Briefly, target cells were re-plated in CAR-T cell media in white-walled 96-well plates, followed by the addition of CAR-T cells at the indicated E:T ratios. Finally, cytotoxicity was calculated based on LDH release using the following formula: Cytotoxicity (%) = [LDH^E:T^- LDH^E^]/LDH^Max^× 100%.

Considering high-heterogeneity of MDSCs, NKG2D CAR-T to MDSC cytotoxicity was assessed by CFSE/7AAD staining. Purified MDSCs were labeled by 0.5μM CSFE for 15 minutes at 37°C, then co-cultured with CAT-T cells at E:T ratios=1:1. Dead MDSCs were as CSFE^+^ and 7AAD^+^.

### 2.6 Cytokine Membrane Array

Cytokine membrane arrays (RayBio, AAM-CYT-3) were performed to determine cytokines expressed in orthotopic and heterotopic tumor lysates. Briefly, tumor tissues were homogenized for extraction of tissue lysate, followed by 10,000g centrifugation for 10 minutes and collection of the supernatants. Notably, the protocol was performed and analyzed according to the manufacturer’s instructions. Cytokines were individually mapped and both background and positive control values were marked.

### 2.7 Isolation and Identification of Myeloid-Derived Suppressor Cells (MDSCs)

MDSCs were isolated from bone marrow cells using a negative magnetic assisted cell sorter (MACS), according to the manufacturer’s instructions (StemCell, 19867). In brief, a suspension of murine bone marrow cells was obtained by repeated aspiration. RBCs were subsequently removed by osmotic lyses. Cells were then incubated with an FcR blocker and antibody cocktail at 4°C for 10 minutes. Finally, MDSCs were purified *via* negative selection using MACS columns. MDSC subsets were identified *via* FACS by staining Gr1 (Biolegend, 108448) and CD11b (Biolegend, 301352). Rae1 expression in MDSCs was measured by confocal laser scanning microscopy.

### 2.8 Quantification of Tumor Growth

Panc02 cells with luciferase knock-in were gifted by Dr. Yan Gu from the Naval Medical University. Orthotopically implanted tumors were monitored by PerkinElmer IVIS Spectrum. Mice were i.p. injected with D-Luciferin (150 mg/kg). The bioluminescent signal was acquired 15 minutes after D-Luciferin injection. During the acquisition procedure, mice were anesthetized with sevoflurane. The *in vivo* relative bioluminescence signal was calculated by Living Image software (PerkinElmer) as recommended by the manufacturer. Heterotopically implanted tumors volume was calculated according to the following formula: Tumor volume = (width)^2^ × (length)/2.

### 2.9 Statistical Analyses

All statistical analyses were performed using GraphPad Prism 8.0. A two-tailed unpaired student t-test was used to determine significance. One-way analysis of variance (ANOVA) with a Bonferroni post-test was used to compare differences among multiple groups. Survival analysis was performed by Kaplan-Meier survival analysis.

## 3 Results

### 3.1 Development of Modified Orthotopic PDAC Model *via* Tumor Slice Transplantation to Minimize Leakage-Related Intra-Abdominal Planting and Support Reliable Tumor Growth

**Figure 1:**
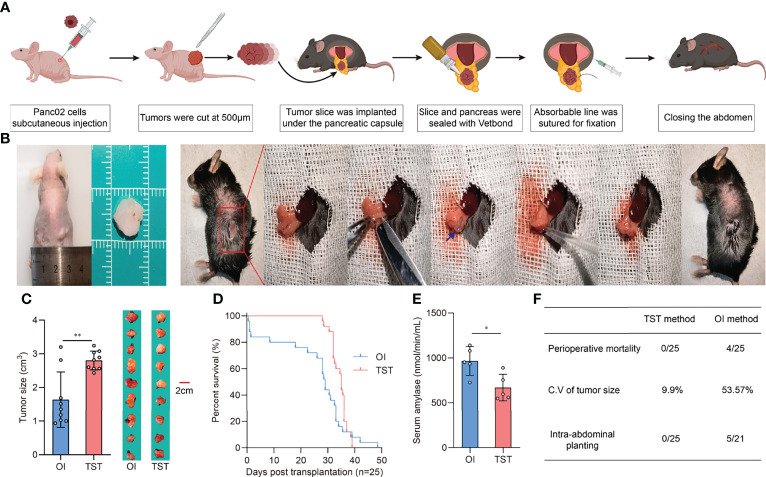
Development and evaluation of the TST mice model. **(A)** Schematic outline of operation procedure. **(B)** Tissue slices of subcutaneous tumor in nude mice (Left). Representative images of the TST mice model construction process (Right). The blue arrow indicates tumor tissue slices. **(C)** Tumor sizes of OI and TST mice models at 21 days post-inoculation (n=9 each, **P<0.01). **(D)** Survival of OI and TST mice models (n=25 each). **(E)** Serum amylase levels of OI and TST mice models at 12 hours post-inoculation (n=5 each, *P<0.05). **(F)** Comparison of necropsy results between OI and TST mice models.

Given that the pancreas of mice is a very small organ (only a few millimeters thick), conventional orthotopic injections (OIs) require an experienced operator to avoid the needle exceeding the tissue which could lead to intra-abdominal dissemination of the tumor suspension. In addition, no matter how precisely the tumor cells are injected into the pancreas, there is no way to prevent the tumor “seeds” from leaking out of the injection port into the peritoneal cavity and causing intra-abdominal planting. Another problem with OI is that the suspension squeezes the pancreatic tissue and causes local inflammation. Perioperative pancreatitis can reduce the amount of the tumor “seeds”, thereby leading to inconsistent tumor volume and causing unexpected death in mice. Therefore, we used tumor slice grafts to develop a new orthotopic model for pancreatic cancer based on previous studies (17, 18). Briefly, the pre-formed tumor was uniformly sliced and used as a “seed”, which was then sutured with an absorbable thread to form a tumor in the tail of the mice pancreas (**Figures 1A, B**). Herein, we established and evaluated PDAC models using the TST and OI methods, respectively. Tumor sizes were then measured in sacrificed recipient mice at 21 days post-inoculation. Results showed that the TST method formed larger orthotopic tumor sizes than the OI method but had a smaller coefficient of variation (9.9% vs. 53.57%), with the TST method exhibiting more stable growth kinetics (**Figure 1C**). Furthermore, according to the survival data of both models, the TST mice died centrally between day 30 and day 40 of the observation period, whereas the OI mice died at a more dispersed time (**Figure 1D**). Another significant difference between the two models was that a small proportion of OI mice experienced perioperative death (survival time <48 h) (**Figure 1D**), which may be attributed to pancreatitis triggered by operation. To test this idea, we examined serum amylase levels in recipient mice at 12 h after inoculation, with results showing that serum amylase levels were lower in TST mice (**Figure 1E**). Meanwhile, gabexate mesylate treatment could reduce the pancreatic damage caused by operation (**Supplementary Figure 1**). In the autopsies of recipient mice, intra-abdominal planting did not occur in all TST mice, but it occurred in 23.8% (5/21) of OI mice (**Figure 1F**). In conclusion, the TST method is a reliable method for developing pancreatic cancer orthotopic mice models.

### 3.2 Orthotopic Graft Shows a Different Immunological Microenvironment Landscape from Heterotopic Graft

**Figure 2:**
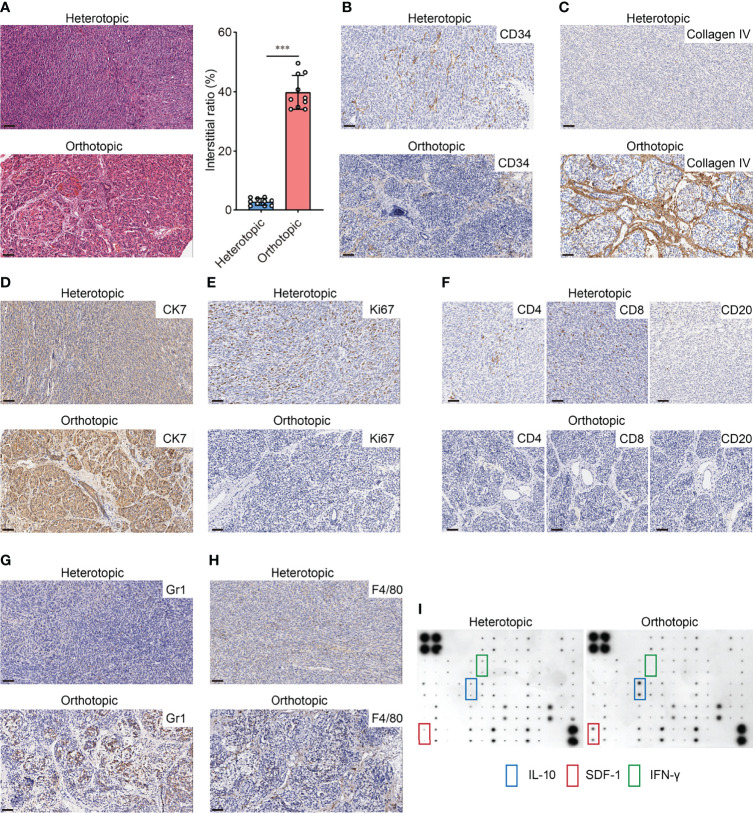
Different immunological microenvironment landscapes of orthotopic and heterotopic graft. **(A)** H&E-stained pancreatic cancer sections (left) and interstitial ratio (right) calculated by ImageJ of orthotopic and heterotopic grafts (n=10 each, ***p<0.001). Scale bar=50μm. **(B)** Representative images of CD34 staining in orthotopic and heterotopic grafts. Scale bar=50μm. **(C)** Representative images of Type IV collagen staining in orthotopic and heterotopic grafts. Scale bar=50μm. **(D)** Representative images of CK7 staining in orthotopic and heterotopic grafts. Scale bar=50μm **(E)** Representative images of Ki67 staining in orthotopic and heterotopic grafts. Scale bar=50μm. **(F)** Representative images of CD4, CD8 and CD20 staining in orthotopic and heterotopic grafts. Scale bar=50μm. **(G)** Representative images of Gr1 staining in orthotopic and heterotopic grafts. Scale bar=50μm. **(H)** Representative images of F4/80 staining in orthotopic and heterotopic grafts. Scale bar=50μm. **(I)** Representative cytokine antibody array for orthotopic and heterotopic grafts lysis.

Based on the orthotopic model established by the TST approach, we demonstrated the differences in the immune microenvironment between orthotopic and heterotopic tumor grafts by testing several indicators. Compared to other solid tumors, the outstanding characteristic of pancreatic cancer is its abundant interstitial and poor vascularization. We first used H&E staining to evaluate the differences between orthotopic and heterotopic (subcutaneous) tumors, with results indicating that orthotopic tumors have richer interstitial formation, whereas heterotopic tumors have little interstitial formation (**Figure 2A**). CD34 expression closely correlates with angiogenesis. The IHC results indicated that there was less angiogenesis in orthotopic tumors compared to heterotopic tumors (**Figure 2B**). It should be noted that type IV collagen is highly expressed in clinical samples of PDAC and contributes to continuous cancer cell growth and maintenance of a migratory phenotype (19). The IHC results indicated that type IV collagen is also highly expressed in orthotopic tumor samples (**Figure 2C**). Furthermore, 90% of pancreatic cancers in humans are PDACs derived from the pancreatic ductal epithelium with a typical epithelial-like phenotype. We found that the epithelial marker CK7 was highly expressed in orthotopic tumors compared to heterotopic tumors (**Figure 2D**), which is consistent with the clinical reality. The expression of Ki67 is strongly associated with tumor cell proliferation and growth and is widely used as a proliferation marker in routine pathological investigation. Herein, heterotopic tumors had higher expression of KI67, which indicates that the tumor growth was supported by a specific tumor microenvironment (**Figure 2E**). In this tumor microenvironment, cell-cell and cell-stroma interactions were different from with those in orthotopic tumors.

PDAC is a typical cold tumor which has little immune cell infiltration. We explored the infiltration of lymphocytes in both tumors and the results showed that the orthotopic tumors had less infiltration of CD4^+^ and CD8^+^ T-cells (**Figure 2F**), which is consistent with the cold tumor characteristics. Also, the MDSCs and TAMs have been shown to increase in PDAC tissues and contribute to immunosuppressive pancreatic TME (20, 21). We found more infiltration of MDSCs (Gr1^+^) and macrophages (F4/80^+^) was observed in orthotopic tumors (**Figures 2G, H**). Those cells may participate in the immunosuppressive microenvironment as cytokine array assay indicated that the production of IL-10 and chemokine SDF-1 was significantly higher in orthotopic tumors than in heterotopic tumors. Nevertheless, lower levels of IFN-γ were found in orthotopic tumors (**Figure 2I**). All these features suggest that the immune microenvironment and the malignant phenotype of the tumor differs significantly between orthotopic and heterotopic PDAC models. Therefore, assessment of immunotherapy using different models may lead to completely different conclusions.

### 3.3 Poor Response of Orthotopic Engraftment Tumor to Immune Checkpoint Blockade Therapy

**Figure 3:**
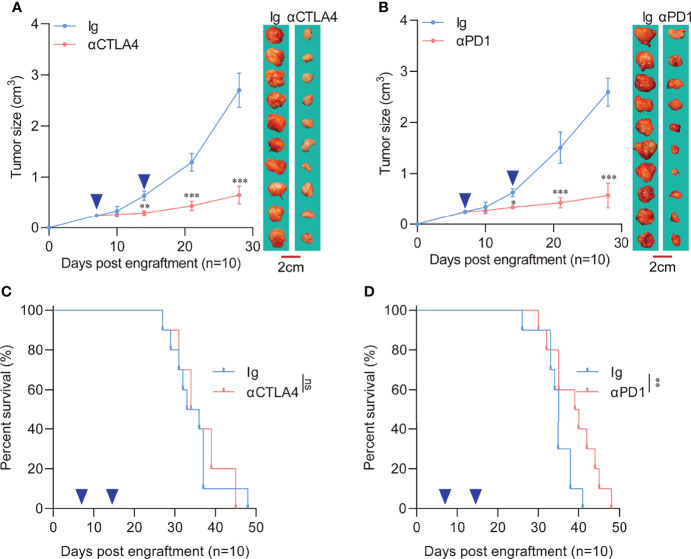
Response of heterotopic or orthotopic engraftment tumors to immune checkpoint blockade therapy. **(A)** Heterotopic tumor-bearing C57 mice were randomly assigned to 2 groups and treated with an anti-CTLA4 antibody (10 mg/kg) or an isotype-matched control antibody. Heterotopic flank tumor size was assayed after treatment. The blue arrows indicate anti-CTLA4 antibody or control Ig administration. (n=10 each, compared with Ig group, **P<0.01, ***P<0.001). **(B)** Heterotopic tumor-bearing C57 mice were randomly assigned to 2 groups and treated with an anti-PD1 antibody (10 mg/kg) or an isotype-matched control antibody. Heterotopic flank tumor size was assayed after treatment. The blue arrows indicate anti-PD1 antibody or control Ig administration. (n=10 each, compared with Ig group, *P<0.05, ***P<0.001). **(C)** Survival of orthotopic tumor recipients after anti-CTLA4 treatment. The blue arrows indicate anti-CTLA4 antibody or control Ig administration (n=10 each, compared with Ig group). **(D)** Survival of orthotopic tumor recipients after anti-PD1 treatment. The blue arrows indicate anti-PD1 antibody or control Ig administration (n=10 each, compared with Ig group, **P<0.01).

A previous meta-analysis demonstrated that treatment of pancreatic cancer patients with immune checkpoint inhibitors did not elicit improvement in either response rates or overall survival (9). The poor performance of PDAC to ICB therapy is likely due to its unique tumor microenvironment. Over the years, the core of PDAC immunotherapy has relied on inducing more intertumoral effector immune cells and reversing immunosuppression (22). Therefore, this study evaluated the responsiveness of the two different tumor engraftment methods to immune checkpoint therapy. Results showed a significant reduction in the heterotopic tumor volume with anti-CTLA4 (**Figure 3A**) or anti-PD1 treatment (**Figure 3B**) compared to the control Ig group. In addition, the orthotopic tumors showed poor response to anti-CTLA4 treatment (**Figure 3C**). Regardless of the extended survival in orthotopic tumor recipients, anti-PD1 treatment did not significantly improve median survival time (34.5 days vs. 35 days) (**Figure 3D**).

### 3.4 Insensitivity of Orthotopic Engraftment Tumor to MUC1 CAR-T Therapy

**Figure 4:**
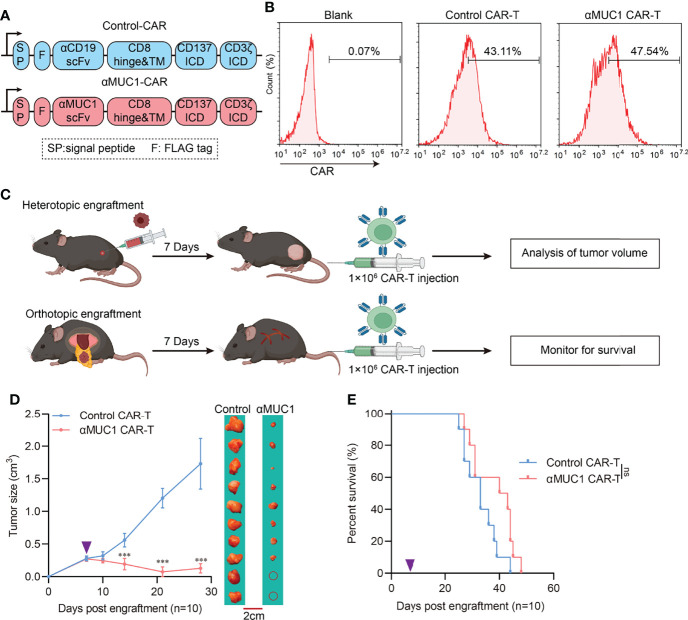
Response of heterotopic or orthotopic engraftment tumor to αMUC1 CAR-T transfer. **(A)** Schematic diagram of CAR structure, VH and VL domains of scFv are joined by a (G_4_S)_3_ linker. Anti-CD19-CAR was used as control. **(B)** Flow cytometry assay of transfection efficiency of CAR-T cells. **(C)** Schematic outline of experimental approaches and analyses. **(D)** Heterotopic tumor size after 1×10^6^ αMUC1 CAR-T transfer. The purple arrows indicate CAR-T cells administration (n=10 each, compared with Control CAR-T group, ***P<0.001). **(E)** Survival of orthotopic tumor recipients after 1×10^6^ αMUC1 CAR-T transfer. The purple arrows indicate CAR-T cells administration (n=10 each, compared with Control CAR-T group).

To further explore the reaction of orthotopic and heterotopic tumors on CAR-T, we constructed a second-generation chimeric antigen receptor with intracellular domain of 4-1BB fused CD3ζ (**Figure 4A**) and transfected into splenic T cells, and then we expanded them to obtain positive >40% CAR-T cells (**Figure 4B**). αMUC1 CAR-T cells killed MUC1^+^ Panc02 cells (**Supplementary Figure 2A**) *in vitro* in a dose-dependent manner but did not kill MUC1^-^ Panc02 cells regardless of the effector-to-target ratio (**Supplementary Figure 2B**). Next, orthotopic and heterotopic implantation models were established, followed by administration of αMUC1-CAR-T after one week (**Figure 4C**). Results showed that the heterotopic tumors had a better response to CAR-T, with 30% of the mice responding completely where the tumors regressed completely (**Figure 4D**). In contrast, the αMUC1-CAR-T treatment failed to significantly prolong the survival of mice in the orthotopic implantation model (**Figure 4E**).

### 3.5 Chimeric NKG2D Receptor Modified T Cells Eliminated MDSCs and Rescued Impaired CAR-T Cell activity in Orthotopic Grafts

**Figure 5:**
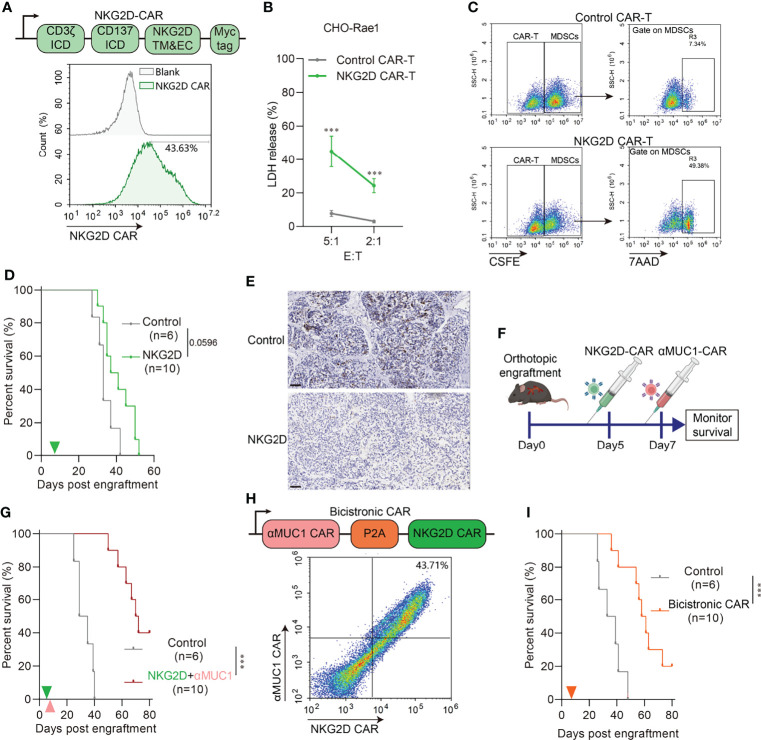
NKG2D CAR-T rescue impaired CAR-T cell activity *via* eliminating MDSCs. **(A)** Schematic diagram of NKG2D-CAR (Upper) and flow cytometry assay of transfection efficiency (Down). **(B)** Cytotoxicity assay of NKG2D CAR-T to Rae1 positive CHO cell at indicated E:T ratio. E:T, effector-to-target ratio. (n=6 compared with Control CAR-T group, ***P<0.001). **(C)** Cytotoxicity assay of NKG2D CAR-T to murine MDSCs at E:T=1:1. MDSCs were pre-labeled by CSFE. CSFE^+^7AAD^+^ cells indicate specific killed MDSCs. **(D)** Survival of orthotopic tumor recipients after NKG2D CAR-T transfer. The green arrows indicate NKG2D CAR-T cells administration. **(E)** Representative IHC images of Gr1 staining in orthotopic tumor 2 days after NKG2D CAR-T transfer. Scale bar=50μm. **(F)** Schematic outline of experimental approaches and analyses. **(G)** Survival of orthotopic tumor recipients after combination of NKG2D CAR-T and αMUC1 CAR-T. The green arrows indicate NKG2D CAR-T cells administration; the orange arrows indicate αMUC1 CAR-T cells administration. (***P<0.001). **(H)** Schematic diagram of bicistronic CAR (Upper) and flow cytometry assay of transfection efficiency (Down). **(I)** Survival of orthotopic tumor recipients after bicistronic CAR-T transfer. The orange arrow indicates CAR-T cells administration (***P<0.001).

We have previously demonstrated that MDSCs are abundantly infiltrated in orthotopic tumors compared to heterotopic tumors. It is well known that MDSCs protect cancer cells from the patient’s immune system, make the tumor resistant to immunotherapy, and allow the tumor to thrive while the patient withers away. Therefore, eliminating MDSCs before CAR-T treatment should improve response rates to cancer therapy and patient survival (23). Although MDSC cells are reported to be a mixed population of cells, they usually express NKG2D ligand on their surface, such as human MICA, MICB, and mouse Rae1 (24). We constructed CHO cells stably expressing Rae1 (**Supplementary Figures 3A, B**) as target. Also, bone marrow MDSCs was isolated by MACS (**Supplementary Figure 3C**) and identified as in **Supplementary Figure 3D**. Rae1 expression on MDSCs was confirmed by confocal microscopy analysis (**Supplementary Figure 3E**). Therefore, we constructed NKG2D-CAR-T (**Figure 5A**) and performed *in vitro* killing assays on Rae1positive cells. Results indicated NKG2D CAR-T could effectively kill Rae1^+^ CHO cells (**Figure 5B**) and MDSCs (**Figure 5C**), but there was no significant type-specific killing activity against Rae1-negative cells (**Supplementary Figure 3F**). *In vivo* experiments demonstrated that NKG2D CAR-T treatment prolonged survival in mice (**Figure 5D**) and effectively eliminated MDSCs from tumors (**Figure 5E**, **Supplementary Figure 4**). In the combination treatment regimen, we administered NKG2D-CAR-T treatment on day 5 of successful TST modeling and αMUC1-CAR-T on day 7 (**Figure 5F**). Results indicated that there was a significantly longer survival in the combination treatment group, with 40% of the mice surviving by the end of the 100-day observation period (**Figure 5G**).

Furthermore, we constructed a bicistronic CAR consisting of a CAR targeting MUC1 linked by the P2A self-cleaving peptide to a NKG2D CAR (**Figure 5H**) and subsequently assayed it in the orthotopic model. Results showed that Bicistronic CAR-T treatment also significantly prolonged the survival of tumor-bearing mice (**Figure 5I**). We next evaluated the capacity of bicistronic CAR-T cells to control more advanced orthotopic models, with the administration on day 15 and 22 post-implantation (**Supplementary Figure 5A**). In the bicistronic CAR-T treated group, 2 out of 6 achieved 80% regression and 3 out of 6 showed complete regression as measured by IVIS imaging (**Supplementary Figures 5B, C**).

## 4 Discussion

Pancreatic cancer, with more than 400,000 new cases annually, is the leading cause of cancer-related death worldwide (25). Complete resection in an early stage remains the only chance for cure, which increases the five-year survival rate from 10% to 25% (26). However, PDAC is characterized by insidious onset and rapid progression, thereby leading to a loss of opportunity for surgery. Studies have revealed that neoadjuvant chemotherapy represented by the FOLFIRINOX regimen can prolong the survival of PDAC patients but drug resistance frequently occurs within one year (27, 28). In recent years, the emergence of ICBs and CAR-T transfer have increased the expectation for immunotherapy against solid cancers, including pancreatic cancer (6, 9). However, although these therapies have been shown to significantly reduce tumor burden in mice models (8, 10), their intervention in clinical application has, so far, rendered little success (29). According to a previous meta-analysis (9), treating PDAC patients with checkpoint inhibitors did not elicit improvement in either response rates or overall survival. Coincidentally, June et al. (10) conducted a phase I study of anti-MUC1 CAR-T in six PDAC patients and found that although there was no effect on the primary PDAC in all patients, liver lesions in one patient showed complete remission. Notably, this phenomenon of different responses to immunotherapy among different tumor sites in the same patient caught our attention and initiated the present study. Given that PDAC’s unique properties are supposed to be inherently linked to the unique physiology and microenvironment of the exocrine pancreas, the heterotopic PDAC model might not be appropriate for evaluation of immunotherapy.

Heterotopic flank tumors are frequently used by researchers because they can be easily and quickly produced and they are approachable for visual monitoring of disease progression and treatment response. Although most studies have not emphasized the anatomical position effect on tumor behavior, some studies still raised concerns regarding this issue. For example, Erstad et al. (14) reported that tumor location influences chemosensitivity to FOLFIRINOX. In addition, a higher level of CpG methylation and accumulation of 3-phosphoglyceric acid were found in orthotopic grafts (30). A reasonable interpretation is that tumor diverse subclones can establish their cooperation in microenvironment remodeling, which allows several dominant clones to exhibit a fitness advantage during tumor progression. Genetic evidence supports this hypothesis to some extent. For instance, Ben-David et al. (12) demonstrated that patient-derived xenografts (PDXs) of various tumors undergo a gradual loss of copy number alterations (CNAs) during *in vivo* passaging. Therefore, these findings suggest that the bias induced by implantation location should be considered when using animal models for the evaluation of tumor treatment.

One of the major obstacles that hampers widespread use of orthotopic models is the challenge of tricky surgical skills and unreliable growth kinetics, especially for PDAC models. Although orthotopic injection of tumor cell suspension is widely used to establish orthotopic models, orthotopic injection of the murine pancreas, which is a thin and narrow organ, is highly susceptible to leakage and consequent intraperitoneal dissemination. Orthotopic injection may also cause intrapancreatic high pressure, which results in vascular compression and duct blockage, ultimately inducing acute pancreatitis and even perioperative death. Even after using Matrigel to restrict the cells disseminated (as Erstad et al. suggested), peritoneal implantation still occurred in 23.8% (vs. 10% as reported) of mice used in this study (14). Therefore, we developed a novel surgical approach for establishing syngeneic PDAC allograft tumor models *via* tumor slice orthotopic transplantation. In contrast to the OI method, the TST method was virtually free of perioperative death and cells disseminated-induced peritoneal implantation. Moreover, TST established models performed reliable growth kinetics with less variability in tumor volume (9.9% vs. 53.57%). These reliable growth kinetics also resulted in more concentrated death time points. This study also raised some operational details in the modeling process: tumor slices should be immersed in ice-cold UW solution, which helps to ensure that the tumor “seeds” have similar viability during surgery. Gabexate mesylate was also administered to inhibit pancreatitis during the perioperative period, which effectively improved the survival rate of the modeling mice.

A previous study reported that the tumor microenvironment (TME) is the biggest impediment to efficient immunotherapy for pancreatic cancer (31). PDAC has a particularly abundant stroma and poor vascularization compared to other solid tumors (32). This study also found abundant interstitial in the orthotopic model, up to about 40% of the total volume, which was absent in the heterotopic model. It should be noted that PDAC accounts for more than 90% of all pancreatic cancers (25) and the epithelial marker (CK7) is usually highly expressed in PDAC samples. Herein, it was that found CK7 had a strong positive expression in orthotopic tumors and low expression in heterotopic tumors, which indicates that the orthotopic tumor bears greater similarity to human pancreatic cancer. Besides, orthotopic tumors exhibited high expression of the suppressive cytokine (IL-10), and infiltration of Gr1^+^CD11b^+^ MDSCs and F4/80^+^ macrophages. Currently, PDAC is immunologically “cold” (33, 34). Similarly, the orthotopic grafts in our model could also be considered as being “cold” due to the absence of tumor-infiltrating lymphocytes.

Sensitivity of tumor models to immunotherapy is also associated with the tumor location, probably due to the orthotopic microenvironment, in which tumors exhibited a low response to immune checkpoint blockades (both PD1 and CTLA4 blockade). This can be confirmed by the lack of responsiveness of pancreatic cancer to ICB mono-therapy in clinical trials (9). Accumulating evidence also demonstrates that the immune checkpoint blockade is effective mainly in tumors with existing T-cell infiltration (35). However, T cell intratumor infiltration is scarce in orthotopic tumors, and poor vascularization and abundant interstitial of orthotopic graft pose barriers to the migration of T-cells into the tumor. Moreover, high expression of suppressive soluble factors within the microenvironment may cause severe T-cell exhaustion (36). Many relevant studies have focused on converting cold tumors into hot tumors to mediate infiltrated T cell activation as a considerable option to solving this rate-limiting step. For example, a preclinical trial indicated that TLR9 agonists (ODN1826 or MGN1703) could significantly enhance the efficacy of ICBs in cold tumors (37). Local radiation therapy can also help turn some cold tumors “hot”. A phase 2 clinic trial showed that radiotherapy improved the efficacy of PD1 monoclonal antibody pembrolizumab in patients with pancreatic cancer (38).

Adoptive cell transfer (ATC) refers to harvesting and *ex vivo* expansion of the patient’s own lymphocytes (39). Compared to ICB therapy, ATC can induce activation of immune cells and perform genetic modification during *ex vivo* culture, thereby increasing the cytotoxic activity, which makes them less dependent on host immune status. CAR-T cell therapy, the most applied therapy in clinical practice of ACT therapies, has exhibited effective function in the elimination of relapsed/refractory B-cell lymphoid malignancies. Currently, there is only a handful of clinical studies on CAR-T for PDAC treatment. Published data from several clinical trials (NCT02465983, NCT01583686, NCT02706782, NCT01935843, and NCT01897415) has demonstrated that PDAC exhibits a poor response to CAR-T therapy, which implies that the orthotopic microenvironment is a significant barrier to CAR-T efficacy. It is worth mentioning that MDSCs play an important role in the immunosuppressive microenvironment of PDAC by mediating T cell dysfunction through multiple pathways, including expression of co-suppressive molecules and secretion of inhibitory cytokines and metabolites (29, 40).Specifically, αMUC1 CAR-T significantly reduced tumor volume in heterotopic tumors lacking MDSC infiltration, with even complete clearance of tumor cells in 20% of the hosts. Therefore, elimination of MDSCs in orthotopic tumors may be an effective strategy to improve CAR-T efficacy. This is consistent with a previous study which proved that MDSCs clearance using Gr1 antibodies effectively improved the CAR-T responsiveness (41).

The NKG2D ligands (NKG2DLs) family is very complex, with several known gene loci encoding slightly different molecules. Notably, NKG2DLs are usually expressed on the cell surface of a variety of tumors and MDSCs (24). In humans, the NKG2DL includes MICA/B and UL16-binding proteins 1-6 (ULBP1-6), whereas in mice it is mainly present as Rae-1 (42). Their receptor NKG2D, expressed on the NK cell surface, promotes cytotoxic lysis of cancer cells expressing NKG2DLs (24). Parihar et al. (23) found that chimeric NKG2D (NKG2D-CD3ζ)-modified NK cells can effectively clear MDSCs in solid tumors and then rescue impaired CAR-T cell activity. In this study, we constructed NKG2D chimeric receptor (NKG2D-41BB-CD3ζ) CAR for adjuvant αMUC1 CAR-T treatment. Results revealed that NKG2D-CAR rapidly cleared MDSCs from orthotopic tumors after reinfusion and significantly prolonged host survival in combination with αMUC1 CAR-T. In addition, we generated a bicistronic CAR-T, including αMUC1 CAR and NKG2D CAR separated by a P2A element. Treatment with dual targeted bicistronic CAR-T cells also resulted in prolonged survival of orthotopic model mice. In summary, this study has described the construction of a novel orthotopic PDAC model using implantation of tissue slices and discussed resistance to immunotherapy from the perspective of the PDAC microenvironment. Based on the results, it is evident that elimination of MDSCs by NKG2D CAR could rescue the impaired CAR-T cell activity.

## Data Availability Statement

The raw data supporting the conclusions of this article will be made available by the authors, without undue reservation.

## Ethics Statement

The animal study was reviewed and approved by Scientific Investigation Board of Navy Medical University.

## Author Contributions

YL, MG, and JZ designed this study and supervised the research. YL and MG wrote the manuscript and contributed in improvement of language and artworks. JW contributed in modelling and *in vivo* experiments. XL and JJ contributed in pathological examination, *in vitro* experiments and analysed data. JL, YZ, and XZ contributed in technical support. All authors contributed to the article and approved the submitted version.

## Funding

This work was supported by the National Natural Science Foundation of China (No. 82172837, No. 82071799, No. 81972683), Shanghai Science and Technology Development Funds (No. 20ZR1469900) and QiMingXing Project (20QA1409100) of Shanghai Municipal Science and Technology Commission.

## Conflict of Interest

The authors declare that the research was conducted in the absence of any commercial or financial relationships that could be construed as a potential conflict of interest.

## Publisher’s Note

All claims expressed in this article are solely those of the authors and do not necessarily represent those of their affiliated organizations, or those of the publisher, the editors and the reviewers. Any product that may be evaluated in this article, or claim that may be made by its manufacturer, is not guaranteed or endorsed by the publisher.
