# Supplementary material for: Orthotopic and Heterotopic Murine Models of Pancreatic Cancer Exhibit Different Immunological Microenvironments and Different Responses to Immunotherapy
Source: Front Immunol. 2022 Jul 7;13:863346. doi: 10.3389/fimmu.2022.863346 (PMC9302770; doi:10.3389/fimmu.2022.863346)
Supplement: Supplementary file 1 [file DataSheet_1.docx]

Supplementary Material

# Supplementary Figures


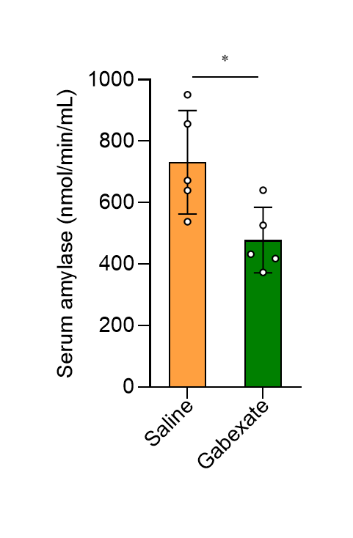


**Supplementary Figure 1**. Serum amylase levels of TST mice models after gabexate mesylate (n=5 each, compared with saline group, *P＜0.05).


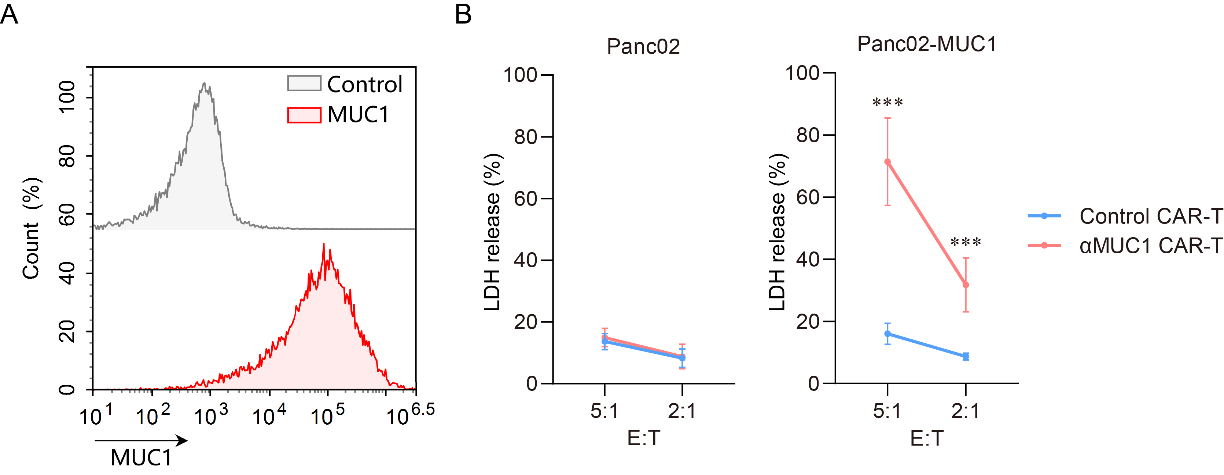


**Supplementary Figure 2.**

A. Flow cytometry assay of MUC1 expression in MUC1 stable expressing Panc02 cells.

B. Cytotoxicity assay to measure αMUC1 CAR-T in Panc02 or MUC1 overexpressed Panc02 cells. αCD19 CAR-T was used as a control.


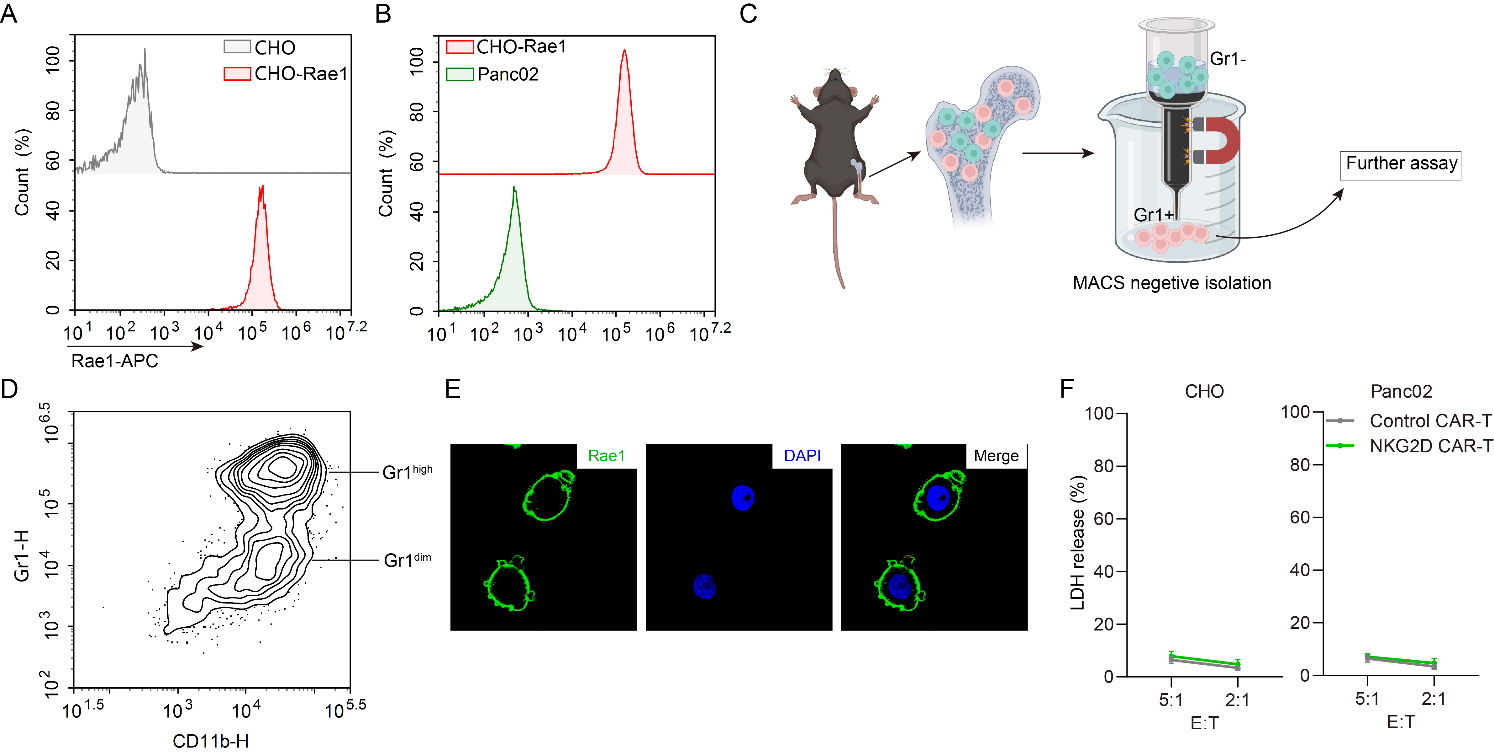


**Supplementary Figure 3.**

A. Flow cytometry assay of Rae1 expression in Rae1 stable expressing CHO cells.

B. Flow cytometry assay of Rae1 expression in Panc02 cells, Rae1 stable expressing CHO cells were used as a positive control.

C. Schematic of isolation of bone marrow MDSCs using Magnetic-Activated Cell Sorting.

D. Characterization of bone marrow mesenchymal stromal cells by flow cytometry.

E. Confocal imaging of Rae1 expression in MDSCs.

F. Cytotoxicity assay of NKG2D CAR-T on Rae1 negative CHO and Panc02 cells at indicated E:T ratio. (n=6 compared with Control CAR-T group).


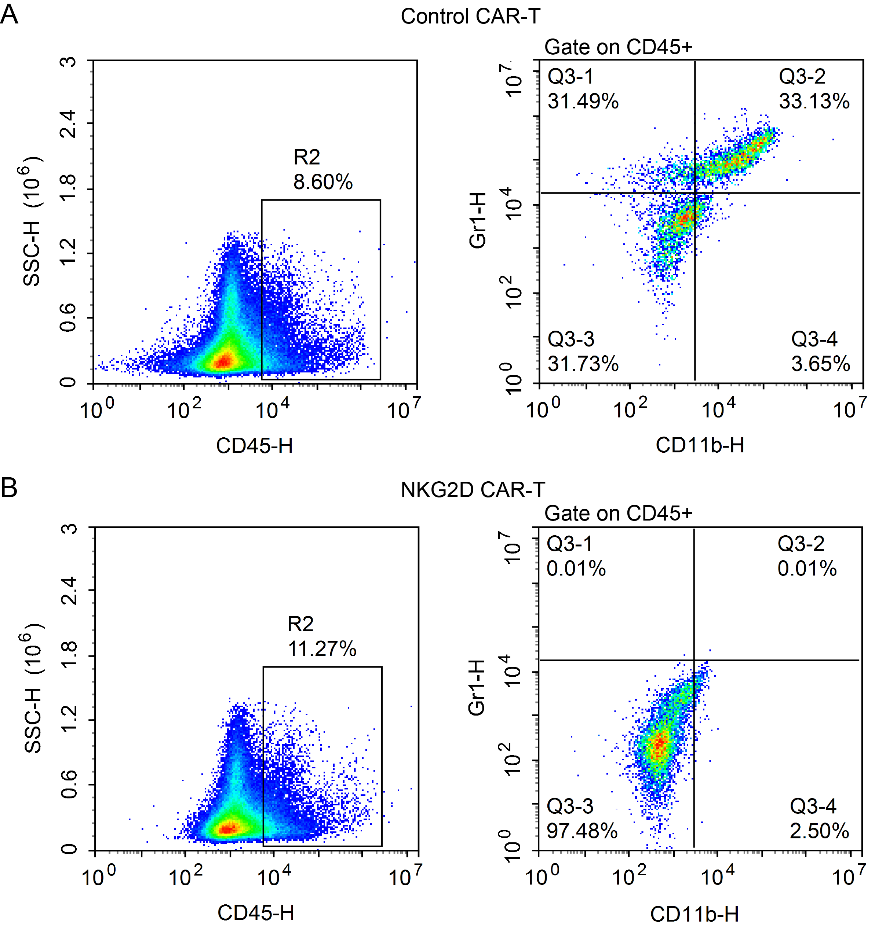


**Supplementary Figure 4.**

FACS plot showing the percentage of Gr1^+^CD11b^+^ cells in the tumour-infiltrating CD45^+^ cells 2 days after NKG2D or control CAR-T cells transfer.


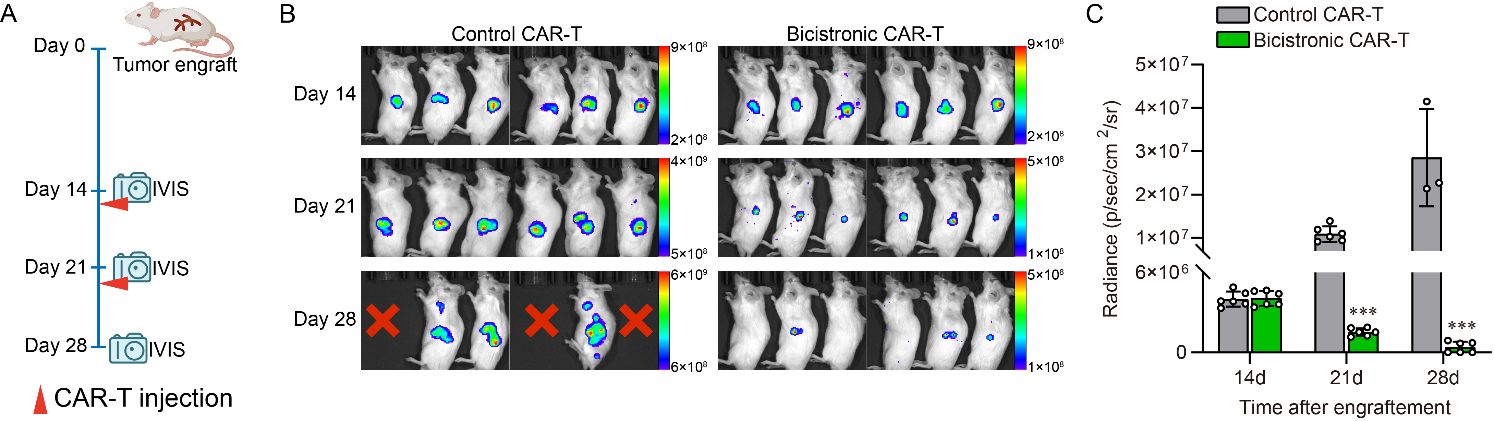


**Supplementary Figure 5.**

A. Schematic work flow for *in vivo* assay bicistronic CAR-T cytotoxicity via IVIS monitoring. Panc02 (luciferase knock-in) tumor slices were orthotopically implanted into NCG mice. On day 14,21 and 28, luciferase signal was detected using IVIS system. 1×10^6^ indicated CAR-T cells were i.v. administrated on day 15 and day 22.

B. Representative IVIS images of mice on day 14, day 21 and day 28. Average radiance intensity was expressed as [p/sec/cm^2^/sr]/[μW/cm^2^].

C. Results of IVIS measurements in the indicated groups. (n=6 compared with Control CAR-T group, ***P＜0.001).
